# Signal Enhancement in Oriented Immunosorbent Assays: A Balance between Accessibility of Antigen Binding Sites and Avidity

**DOI:** 10.3390/bios11120493

**Published:** 2021-12-03

**Authors:** Vanessa Susini, Vanna Fierabracci, Gaia Barria, Lisa Dodoli, Laura Caponi, Aldo Paolicchi, Maria Franzini

**Affiliations:** 1 Department of Translational Research and of New Surgical and Medical Technologies, University of Pisa, Via Savi 10, 56126 Pisa, Italy; vanessa.susini@med.unipi.it (V.S.); vanna.fierabracci@unipi.it (V.F.); gaiabarria.93@gmail.com (G.B.); lisadodoli18@gmail.com (L.D.); laura.caponi@unipi.it (L.C.); aldo.paolicchi@unipi.it (A.P.); 2 Laboratory of Clinical Pathology, Santa Chiara University Hospital, Via Roma 67, 56126 Pisa, Italy

**Keywords:** immunoassay, oriented binding, steric hindrance, antibody avidity, reduced IgG, half-fragment antibodies

## Abstract

The sensitivity of immunoassays was reported to be increased by the orientation of antibodies. We investigated how the size and valence of antigens and orientation and valence of antibodies contribute to the analytical sensitivity of ELISA. Antigens differing in size and number of epitopes were compared using oriented and non-oriented ELISAs: the orientation of antibodies was obtained coating half-fragment antibodies on maleimide microplates, while, in non-oriented ELISA, whole antibodies were randomly physisorbed. The oriented assay performed better than the non-oriented one at each concentration (0.4–3.3 ng/mL) of a small monomeric antigen (cardiac Troponin I, 24 kDa, Rh 3 nm). No significant differences were observed with a large monovalent antigen (prostate-specific antigen-alpha(1) antichymotrypsin, 90 kDa, Rh > 3 nm), since its steric hindrance overcame the increased availability of antigen binding sites given by orientation. Large multivalent antigens (ferritin, 280 kDa, Rh 6 nm; α-fetoprotein, >70 kDa, Rh > 3.3 nm) performed better in non-oriented assays. In this case, the repeated epitopes on the surface of the antigens favored the engagement of both antigen binding sites of the whole IgG, thus suggesting that avidity represented the leading force in this experimental setting. In conclusion, the design of high-sensitivity ELISAs should consider the dimension and valency of antigens in addition to the affinity and avidity of antibodies.

## 1. Introduction

Antibodies were generally used as biological capture molecules in immunoassays as they form specific and high-affinity interactions with their antigens. These assays were widely used in the medical, environmental and life science areas [1]. One of the challenges faced in the design of immunoassays for clinical laboratories is to achieve the highest possible analytical sensitivity. One possible approach to enhance the binding of antigens and, hence, the sensitivity of immunoassays, consists in the homogeneous orientation of capturing antibodies on the surface of ELISA microplates [2,3,4,5,6,7,8]. Indeed, since the early 1990s, researchers investigated how the close proximity to hydrophobic solid phases affects antigen–antibody interactions. Butler and coworkers [9] showed that only 5–10% of the antigen binding sites are effectively available when antibodies are randomly adsorbed on the polystyrene surface, but this percentage could be increased by up to 70% when antibodies are immobilized by biotin-streptavidin linkage. Since then, the described improvements are highly variable, and the contribution of analyte and antibody properties to the sensitivity of oriented assays is poorly defined. Trilling and co-workers [10] showed that the extent of signal enhancement depends not only on the dimensions of antigens but also on the affinity constants of antibodies. They observed that lower-affinity interactions benefit more from a uniform orientation than stronger ones. Peluso et al. [11] observed that the number of available binding sites on the capturing surface was also involved in the signal enhancement, thus introducing the role of avidity in the development of immunoassays. Avidity is a measure of the overall strength of an antibody–antigen complex, and it mainly depends on the affinity and valency of both antigens and antibodies [12]. Affinity, instead, is a measure of the strength of interaction between an epitope and a single antigen binding site.

In a previous study, we oriented monoclonal anti-free prostate specific antigen (anti-fPSA) antibodies on a conveniently modified surface by reducing the disulphide bridges located in the hinge region of the antibodies. The ELISA assay, performed with the oriented monovalent half fragment antibodies (i.e., reduced IgG, rIgG), showed improved sensitivity compared to the same assay performed with randomly adsorbed anti-fPSA [13]. In the current study, we evaluated the effect of some antigen properties (i.e., size and valence) and the corresponding antibodies (i.e., orientation and valence) on the signal enhancement in oriented or non-oriented immunoassays. Monomeric and multimeric proteins (Table 1) with ascending molecular weight were used as antigens in ELISAs based on randomly adsorbed IgG or oriented ELISAs based on the corresponding monovalent rIgG. The results showed that the leading forces involved in the signal enhancement were different depending on the size and valence of antigens. The oriented immunoassays based on monovalent rIgG provided a significant signal increase with small monomeric antigens due to the greater number of accessible antigen binding sites. For large multimeric antigens, instead, the highest signal enhancement was observed with non-oriented whole antibodies due to their higher avidity compared to rIgG.

## 2. Materials and Methods

### 2.1. Chemicals

Mouse monoclonal anti-alpha-fetoprotein (anti-AFP) antibodies, mouse monoclonal anti-Troponin I (anti-TnI) antibodies, mouse monoclonal anti-ferritin (anti-Ferr) antibodies, mouse monoclonal anti-PSA-alpha(1)-antichymotrypsin (anti-PSA-ACT) antibodies, VIDAS^®^ AFP assay ref. 30,413 VIDAS^®^ TNHS assay ref. 415,386, VIDAS^®^ Ferritin assay ref. 30,411, VIDAS^®^ TPSA assay ref. 30,428 and VIDAS^®^ Optical Substrate were supplied by bioMérieux Italia (Bagno a Ripoli, Italia). Mouse monoclonal secondary antibodies were obtained from the corresponding VIDAS^®^ kits. Generally, these antibodies are directed against epitopes that differ from those recognized by capture antibodies. The only exception was the secondary anti-ferritin antibody, which is the same as the capturing antibody, since ferritin consists of 24 repeated subunits.

Sephadex G-25 in PD-10 Desalting Columns (GE Healthcare, Little Chalfont, UK), 2-Mercaptoethylamine (2-MEA), ethylenediaminetetraacetic acid disodium salt (EDTA), Ca^2+^ and Mg^2+^ free Dulbecco’s Phosphate Buffered Saline (PBS), anhydrous L-cysteine chlorohydrate (L-Cys •HCl) were purchased from Sigma Chemical Co. (St. Louis, MO, USA).

Pierce™ Maleimide Activated Plates (Black) were purchased from Thermo Fisher Scientific (Waltham, MA, USA), while Bio-Plex Pro™ Flat Bottom Plates (black) were acquired from Bio-Rad (Hercules, CA, USA). All solutions were prepared using bi-distilled water (ddH_2_O) and all reagents were used without further purification.

### 2.2. Antigens

In this study, we analyzed both monovalent and multivalent antigens of different molecular weights. The monovalent ones were cTnI and PSA-ACT, the multivalent AFP and ferritin.

cTnI was a 23.9 kDa monomeric rod-shaped protein consisting of 209 amino acids with a Stokes radius of 3 nm [14] (Supplementary Material, Figure S1).

PSA was a serine protease of 30 kDa; its most abundant immunoreactive form was the 90 kDa complex PSA-ACT. Anti-PSA-ACT antibodies recognize an epitope exposed on the PSA moiety of the complex [15,16]; thus, PSA-ACT can be considered a large monovalent antigen with Stokes radius >3 nm (Supplementary Material, Figure S2).

AFP is a globular protein of about 70 kDa with Stokes radius of 3.26 nm; it can associate in dimers, trimers and even tetramers that are immunologically indistinguishable [17,18]. Considering this, AFP was considered a large multivalent antigen (Supplementary Material, Figure S3).

Finally, ferritin was selected as a large multimeric antigen, since it is a globular heterodimeric protein of 474 kDa constituting 24 subunits and a hydrodynamic radius of 6 nm [19] (Supplementary Material, Figure S4). Thus, ferritin was selected as a second large multivalent antigen.

The S1 calibrators from the corresponding commercial kits for the VIDAS^®^ system were used as an antigen source for ELISA experiments (see chemical list).

### 2.3. Antibody Reduction Using 2-Mercaptoethylamine (2-MEA)

Antibodies were reduced according to the previously described protocol [13]. In brief: 1 mg/mL antibodies were reduced by 53 mM 2-Mercaptoethylamine (2-MEA) in PBS with 10 mM EDTA (PBS-EDTA). The reduction mixtures were incubated for 90 min at 37 °C under mild agitation. Then, the reducing agent and EDTA were removed using a Sephadex G-25 in PD-10 Desalting Column according to the manufacturer’s instructions.

The presence of rIgG (75 kDa) was assessed by non-reducing 8% SDS-PAGE (polyacrylamide gel electrophoresis); protein bands were stained with Coomassie Blue.

### 2.4. Native PAGE

The presence of AFP aggregates was evaluated by native 10%-PAGE. Protein sample (5 μL) were diluted in 15 μL ddH_2_O and sample buffer 5× (0.175 M Tris HCl pH 6.8, 35% glycerol and 0.05% bromophenol blue). Resolving gels without SDS were run at 150 V in native running buffer (0.025 M TRIS-Base pH 8.3, 0.192 M Glycine). Protein bands were stained with Coomassie Blue.

### 2.5. ELISAs Performed with Oriented or Not Oriented Antibodies

The antigen binding capacity of oriented or physisorbed antibodies was tested on maleimide-activated or polystyrene microplates, respectively. Polystyrene or maleimide activated microplates were rinsed with 200 µL of PBS containing 0.01% (*w*/*v*) TWEEN^®^ 20 (PBS-T) and then coated with 50 µg/mL whole or reduced antibodies in PBS-EDTA (100 µL). For maleimide-activated microplates, the manufacturer suggests 1–50 µg/mL sulfhydryl-containing peptide for the coating step. In a preliminary assay, 10 µg/mL, 25 µg/mL and 50 µg/mL rIgG were tested by ELISA using four concentrations of antigen; the ELISA procedure was performed according to the manufacturer’s instructions. The most reproducible results were obtained using 50 µg/mL rIgG (data not shown). After 2 h incubation at room temperature, coating solution was removed by three washing steps with PBS-T (200 µL). Nonspecific binding of polystyrene microplates was blocked by 1 h incubation with 5% (*w*/*v*) non-fat milk solubilized in PBS-T (100 µL), and then washed as previously described. Nonspecific binding of maleimide-activated microplates was blocked by 1 h incubation at room temperature with freshly prepared 10 µg/mL cysteine in PBS.

Samples were incubated for 1 h at room temperature, then unbound antigens were removed by three washing steps. PBS was used as negative control. Secondary antibodies conjugated with alkaline phosphatase (ALP), available in the VIDAS^®^ kits, were incubated for 1 h at room temperature. After three washing steps, 200 µL/well of ALP fluorescent substrate (VIDAS^®^ Optical Substrate) was added. The generated fluorescence was measured after 20 min incubation time on Fluoroskan Ascent plate reader (Thermo Fisher Scientific. Waltham, MA, USA) with 20 msec integration time, excitation wavelength at 390 nm and emission at 460 nm. All incubation steps were carried out at room temperature.

The best equation describing the dependence of the signal value on the concentration was Padé (1,1) equation: Y = (A0 + A1 × X)/(1 + B1 × X). Nonlinear fit analysis was performed with the software Graph Pad Prism 9.

## 3. Results

### 3.1. Antibodies Reduction

**Figure 1:**
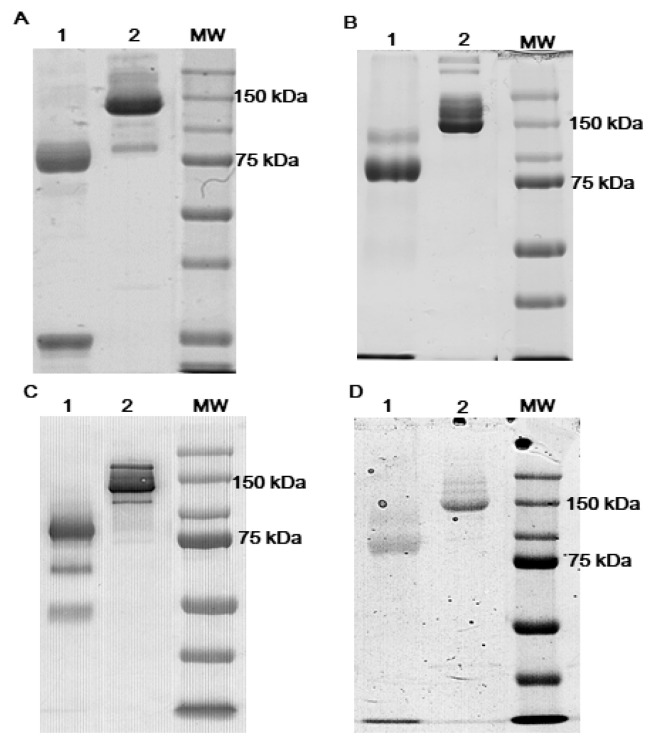
Non-reducing SDS-PAGE of reduced antibodies: anti-cTnI (**A**), anti-FERR (**B**), anti-AFP (**C**) and anti-fPSA-ACT (**D**). Lanes 1, reduced antibodies; lanes 2, whole antibodies. cTnI, cardiac troponin I; FERR, ferritin; AFP, alpha-fetoprotein; PSA-ACT, prostate-specific antigen-alpha(1)-antichymotrypsin.

All the tested antibodies, anti-FERR, anti-cTnI anti-AFP and anti-PSA-ACT, were effectively reduced, as shown by the band at 75 kDa, corresponding to the molecular weight of a rIgG (Figure 1, lanes 1).

### 3.2. Signal Enhancement in Oriented ELISAs Using Monomeric Antigens

The selected monovalent antigens cTnI and PSA-ACT were used to evaluate the contribution of steric hindrance of the antigen to the signal enhancement in oriented ELISAs. Maleimide activated microplates were used to bind rIgG through the free sulfhydryl groups of the hinge region, thus obtaining an oriented layout of antibodies: the results obtained in this setting were compared to those collected with randomly physisorbed whole antibodies on polystyrene microplates.

#### 3.2.1. cTnI

**Figure 2:**
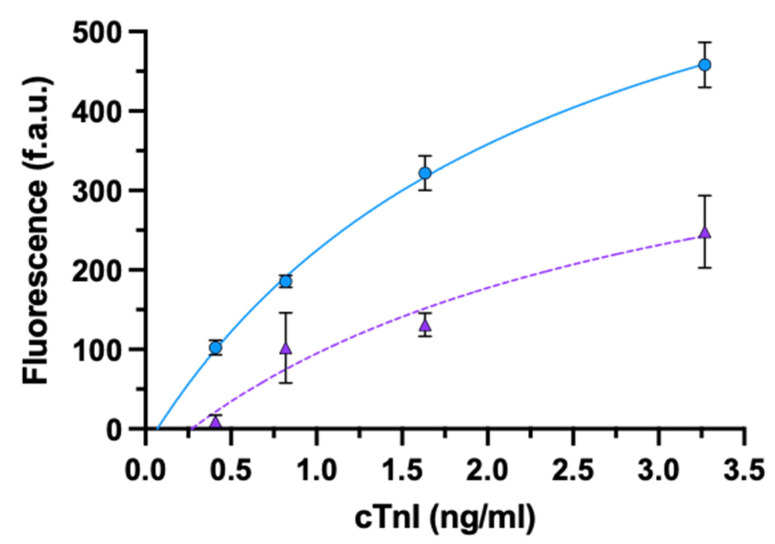
ELISA for cTnI using oriented rIgG (circle) or non-oriented whole-IgG (triangle). Data are presented as mean ± SD (*n* = 4). Fitting equations: rIgG (solid line) y = (−23.75 + 343.9x)/(1 + 0.47x); IgG (dashed line) y = (−47.86 + 177.10x)/(1 + 0.36x).

We tested four different concentrations of cTnI antigen. The signal obtained using oriented rIgG was about ten-fold higher than that obtained with non-oriented whole IgG (mean ± SD: 9.45 ± 7.84; 102.30 ± 9.21, respectively) for the lowest concentration of cTnI (0.41 ng/mL). For the other concentrations, the signal enhancement was about two-fold (Figure 2, Table S1).

#### 3.2.2. PSA-ACT

**Figure 3:**
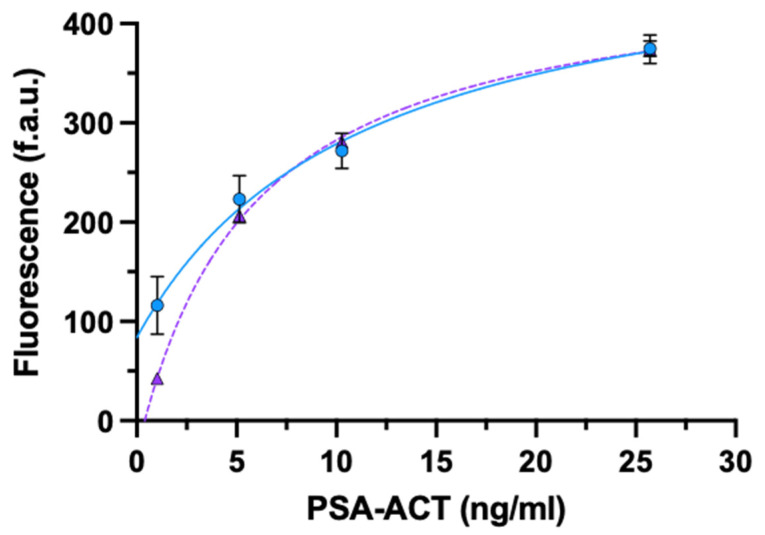
ELISA for the detection of PSA-ACT using oriented rIgG (circle) or non-oriented whole-IgG (triangle). Data are presented as mean ± SD (*n* = 4). Fitting equations: rIgG (solid line) y = (83.74 + 44.24x)/(1 + 0.09x); IgG (dashed line) y = (−32.50 + 81.26x)/(1 + 0.18x).

We tested four concentrations of PSA-ACT; the signals generated by oriented rIgG were compared to those of randomly adsorbed whole IgG (Figure 3, Table S2).

For the lowest concentration tested (1.03 ng/mL), rIgG showed 3-fold signal enhancement compared to whole IgG (mean ± SD: 116.01 ± 29.21; 42.61 ± 1.19, respectively), while there were no significant differences between the signal generated by oriented and randomly adsorbed antibodies for the other analyte concentrations.

### 3.3. Signal Enhancement in Multimeric Antigens

The multivalent antigens AFP and ferritin are sufficiently large to neutralize the signal improvement associated with antibody orientation [10,20]. For that reason, we selected them as models to evaluate the effect of avidity on ELISAs sensitivity.

As before, four different concentrations of AFP and ferritin were tested in ELISAs based on rIgG (monovalent) oriented on maleimide-activated microplates, or whole IgG (bivalent) randomly adsorbed on polystyrene microplates.

#### 3.3.1. AFP

**Figure 4:**
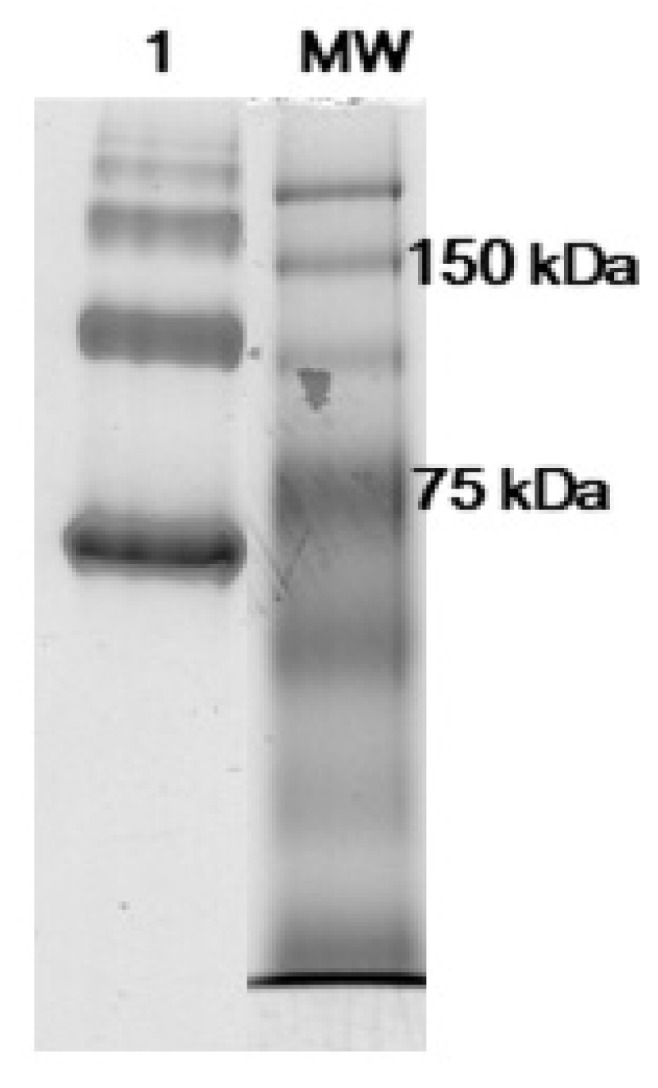
NATIVE-PAGE of calibrator S1 of VIDAS^®^ AFP. MW, molecular weights.

Purified AFP tends to aggregate in multimers that are immunologically indistinguishable from the monomer [18]. Native PAGE was used to characterise the presence of aggregates within the calibrator S1 of VIDAS^®^ AFP. Native PAGE showed the presence of four bands with an electrophoretic mobility that is compatible with AFP monomers (70 kDa), dimers (140 kDa), trimers (210 kDa) and tetramers (280 kDa) (Figure 4).

**Figure 5:**
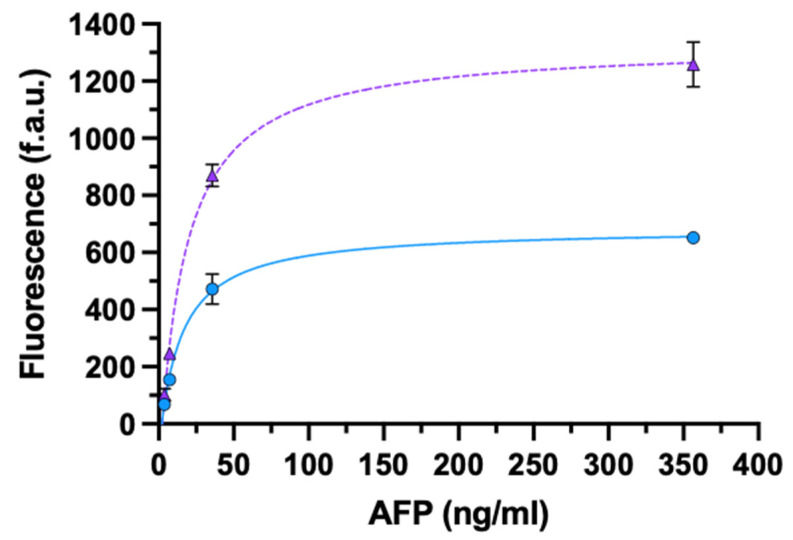
ELISA for the detection of AFP using oriented monovalent rIgG (circle) or non-oriented bivalent IgG (triangle). Data are presented as mean ± SD (*n* = 4). Fitting equations: rIgG (solid line) y = (−98.43 + 49.05x)/(1 + 0.07x); IgG (dashed line) y = (−187.6 + 81.51x)/(1 + 0.06x).

The results obtained with AFP are shown in Figure 5. In this case, no or slight differences were observed at the two lowest concentrations of AFP, while the signal generated with a whole bivalent IgG assay was about twice that obtained with monovalent rIgG at the two highest concentrations tested (Table S3).

#### 3.3.2. Ferritin

**Figure 6:**
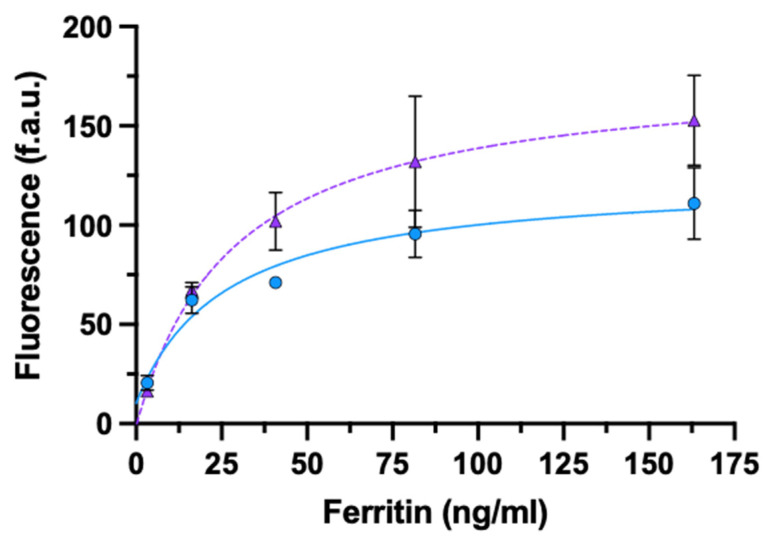
ELISA for the detection of ferritin using oriented monovalent rIgG (circle) or non-oriented bivalent IgG (triangle). Data are presented as mean ± SD (*n* = 4)**.** Fitting equations: rIgG (solid line) y = (10.32 + 4.75x)/(1 + 0.04x); IgG (dashed line) y = (−1.26 + 6.30x)/(1 + 0.04x).

The results collected on both whole-IgG and rIgG-based ELISAs using ferritin as antigen are summarized in Figure 6. As for AFP, no significant differences were observed between whole-IgG or rIgG-based assays at the two lowest concentrations of ferritin, while the whole-IgG setting performed better than rIgG for increasing ferritin concentrations (Table S4).

## 4. Discussion

The results highlighted that the orientation of antibodies provided the maximum improvement with small monomeric analytes, such as cTnI. Instead, the orientation showed only a slight enhancement with large antigens such as PSA-ACT, since the accessibility to the antigen binding sites was limited by the steric hindrance of the analyte [10,11]. On the contrary, the rIgG approach showed no advantages when applied to multivalent antigen.

The results obtained with cTnI were in agreement with those observed using free-PSA (fPSA), an antigen with the same Stokes radius of cTnI (≈3 nm); thus, their steric hindrance on the capturing surface was similar [13]. For fPSA, the signal improvement obtained with oriented ELISAs decreased with increasing antigen concentration.

The monovalent nature of cTnI allowed us to appreciate the contribution of antibody affinity to the signal enhancement in ELISAs. Our results were comparable to those published by Peluso et al. [11]: these authors showed that the orientation of capturing antibodies provided a maximum advantage (ten-fold increase) when using antigens at concentration below the dissociation constant, while those above the main contribution to the signal enhancement depended on the overall surface binding capacity. We also observed a ten-fold signal enhancement with the lowest concentrations of cTnI in ELISAs performed with rIgG, while, for the other concentrations, the signal improvement decreased by about three-fold. The overall data obtained for cTnI, as well as for fPSA [13], suggested the same conditions described by Peluso [11]. Unfortunately, the affinity and dissociation constants of the anti-cTnI and anti-fPSA antibodies used in our study are not known.

Our results were corroborated by the work of Mackey and coworkers [21], which developed a theoretical model to investigate how the size of the antigens, as well as the orientation and affinity of antibodies, affected the binding in immunoassays. Authors found that the accessibility of the antigen-binding sites, rather than the affinity of antibodies, affected the strength of the signal.

PSA-ACT, a large monovalent antigen, allowed us to investigate how the steric hindrance of the antigen influences the binding capacity of the capturing surface in ELISAs. In this case, the signal enhancement provided by the oriented rIgG was immediately lost at increasing concentrations of PSA-ACT when the antigen steric hinderance overtook the advantage of antibody orientation. In these conditions, the higher number of accessible antigen binding sites provided by rIgG became irrelevant in comparison with the non-oriented capturing surface.

The two multimeric studied antigens, ferritin and AFP, allowed us to face another aspect of antigen–antibody binding properties. Their multivalence emphasized the role of avidity in ELISAs. The repeated epitopes on the surface of ferritin and AFP favored the engagement of both antigen-binding sites of whole IgG, while the role of orientation become negligible due to the steric hindrance. These results suggested that avidity, which measures the total strength of binding between an antibody and its antigen, was the leading force involved in the binding of multimeric analytes. These results agreed with the observation of Bush and Knotts [22]; indeed, the authors, thanks to the developed coarse-grain model, found that multiple binding sites stabilize the immunocomplexes in microarrays.

Trilling and coworkers [10] investigated the role of avidity in oriented immunoassays and they did not found relationships between the valency of antigens and the effect of orientation. This is not in conflict with our results, since, in their experiments, the authors used an oriented or randomly adsorbed variable domain of heavy-chain antibodies of llama, which consists of only monovalent antigen-binding sites.

To conclude, the orientation of antibodies in immunosorbent assays represents an advantage when applied to the detection of small monomeric analytes, especially at antigen concentrations below the dissociation constant of the antibody.

## 5. Conclusions

In this work, we explored the effect of some properties of antigens (size and valence) and antibodies (orientation and valence) on the signal enhancement in oriented or non-oriented ELISA. The results highlighted that, in oriented immunoassays, the higher number of accessible antigen-binding sites provided a significant advantage when using small monomeric antigens. As the size of antigens increases, the orientation did not offer any advantage, since the steric hindrance on the surface limits the accessibility to the antigen-binding sites. Interestingly, in the case of multivalent antigens, the highest signal enhancement was observed with non-oriented whole antibodies, highlighting the role of avidity in immunoassays. Indeed, the repeated epitopes favored the engagement of both antigen-binding sites of whole IgG, while the role of orientation become negligible due to the steric hindrance.

The main limitation of this work is the lack of affinity constants for the antibodies used. Indeed, to corroborate our results, we will determine the association and dissociation constants of both whole and reduced antibodies using the surface plasmon resonance technique. Then, the affinity constants, together with the data disclosed here, will be used for the development of an in silico model to assist researchers and the R&D department of in vitro diagnostic industries in the design of highly sensitive immunosensors based on the target analyte.

## Figures and Tables

**Table 1:** Properties of the antigens used in the study.

Antigens	MW (kDa)	Stokes Radius (nm)	Epitope	Epitope Number
cTnI	24	3	N-terminus	1
PSA-ACT	90	>3	n.a.	1
AFP	70	3.26	n.a.	1–4
Ferritin	474	6	n.a.	12

cTnI, cardiac troponin I; PSA-ACT, prostate specific antigen-alpha**(1)** antichymotrypsin; AFP, alpha-fetoprotein.

## Data Availability

Raw data are available in Supplementary Material.

## Supplementary Materials

The following are available online at https://www.mdpi.com/article/10.3390/bios11120493/s1. Table S1. ELISA for the detection of cTnI using non-oriented whole-IgG or oriented rIgG. Data are represented as mean ± SD (*n* = 4). cTnI, cardiac troponin I. Table S2. ELISA for the detection of PSA-ACT using non-oriented whole-IgG or oriented rIgG. Data are represented as mean ± SD (*n* = 4). PSA-ACT, prostate-specific antigen-alpha(1)antichymotrypsin Table S3. ELISA for the detection of AFP using non-oriented whole-IgG or oriented rIgG. Data are represented as mean ± SD (*n* = 4). AFP, alpha-fetoprotein. Table S4. ELISA for the detection of ferritin using non-oriented whole-IgG or oriented rIgG. Data are represented as mean ± SD (*n* = 4). Figure S1. Core domain of human cardiac troponin. Cardiac troponin I (red) is a rod-shaped protein of 23.9 kDa and Stokes radius of 3 nm. In blue troponin C and in green troponin T. PDB accession code 1J1D [23]. Figure S2. Prostate-specific antigen (PSA) in white complexed with alpha(1)-antichymotrypsin (ACT) in blue [15]. Figure S3. Alpha-fetoprotein (AFP) monomer structure model. AFP is a V-shaped protein of about 70 kDa with Stokes radius of 3.26 nm [24]. Figure S4. Cartoon representation of Ferritin, a globular heterodimeric protein of 474 kDa with hydrodynamic radius of 6 nm. PDB accession code 1FHA [25].
