# Supplementary material for: Signal Enhancement in Oriented Immunosorbent Assays: A Balance between Accessibility of Antigen Binding Sites and Avidity
Source: Biosensors (Basel). 2021 Dec 3;11(12):493. doi: 10.3390/bios11120493 (PMC8699147; doi:10.3390/bios11120493)
Supplement: Supplementary file 1 [file biosensors-11-00493-s001.zip › biosensors-1456265-supplementary.pdf]

biosensors

MDPI

Communication

Signal Enhancement in Oriented Immunosorbent Assays: A Balance between Accessibility of Antigen Binding Sites and Avidity

Vanessa Susini [1], Vanna Fierabracci [1], Gaia Barria [1], Lisa Dodoli [1], Laura Caponi [1,2], Aldo Paolicchi [1,2] and Maria Franzini [1,2]*

[1] Department of Translational Research and of New Surgical and Medical Technologies, University of Pisa, Via Savi 10, 56126 Pisa, Italy; vanessa.susini@med.unipi.it (V.S.); vanna.fierabracci@unipi.it (V.F.); gaiabarria.93@gmail.com (G.B.); lisadodoli18@gmail.com (L.D.); laura.caponi@unipi.it (L.C.); aldo.paolicchi@unipi.it (A.P.)

[2] Laboratory of Clinical Pathology, Santa Chiara University Hospital, Via Roma 67, 56126 Pisa, Italy; laura.caponi@unipi.it (L.C.); aldo.paolicchi@unipi.it (A.P.)

* Correspondence: maria.franzini@unipi.it

Table S1. ELISA for the detection of cTnI using non-oriented whole-IgG or oriented rIgG. Data are represented as mean ± SD (n = 4). cTnI, cardiac troponin I.

cTnI (ng/ml)	Oriented antibodies (fluorescence a.u.)	Non-oriented antibodies (fluorescence a.u.)
0.41	102.30 ± 9.21	9.45 ± 7.84
0.82	185.42 ± 7.51	102.01 ± 44.18
1.64	321.72 ± 21.8	131.13 ± 14.57
3.27	457.96 ± 28.4	248.21 ± 45.67

Table S2. ELISA for the detection of PSA-ACT using non-oriented whole-IgG or oriented rIgG. Data are represented as mean ± SD (n = 4). PSA-ACT, prostate-specific antigen-alpha(1)antichymotrypsin

PSA-ACT (ng/ml)	Oriented antibodies (fluorescence a.u.)	Non-oriented antibodies (fluorescence a.u.)
1.03	116.01 ± 29.21	42.61 ± 1.19
5.14	223.15 ± 23.56	206.35 ± 3.39
10.28	271.93 ± 17.91	280.44 ± 3.71
25.70	374.76 ± 7.78	374.11 ± 14.50

Table S3. ELISA for the detection of AFP using non-oriented whole-IgG or oriented rIgG. Data are represented as mean ± SD (n = 4). AFP, alpha-fetoprotein.

AFP (ng/ml)	Oriented antibodies (fluorescence a.u.)	Non-oriented antibodies (fluorescence a.u.)
3.57	68.07 ± 7.24	102.16 ± 21.41
7.13	154.84 ± 5.60	246.58 ± 18.74
35.66	472 .10 ± 52.44	870.03 ± 38.46
356.55	652.02 ± 8.25	1257.82 ± 78.43

Table S4. ELISA for the detection of ferritin using non-oriented whole-IgG or oriented rIgG. Data are represented as mean ± SD (n = 4).

Ferritin (ng/ml)	Oriented antibodies (fluorescence a.u.)	Non-oriented antibodies (fluorescence a.u.)
3.26	20.55 ± 3.65	16.37 ± 2.83
16.32	62.20 ± 6.96	67.32 ± 3.94
40.80	71.13 ± 2.39	101.88 ± 14.48
81.60	95.65 ± 11.91	131.88 ± 33.08
163.2	110.87 ± 18.07	152.78 ± 22.63

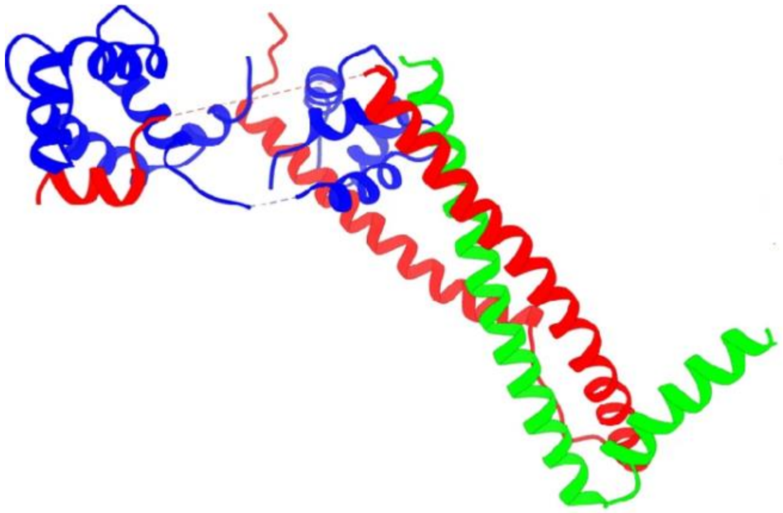

Figure S1. Core domain of human cardiac troponin. Cardiac troponin I (red) is a rod-shaped protein of 23.9 kDa and Stokes radius of 3 nm. In blue troponin C and in green troponin T. PDB accession code 1J1D [23].

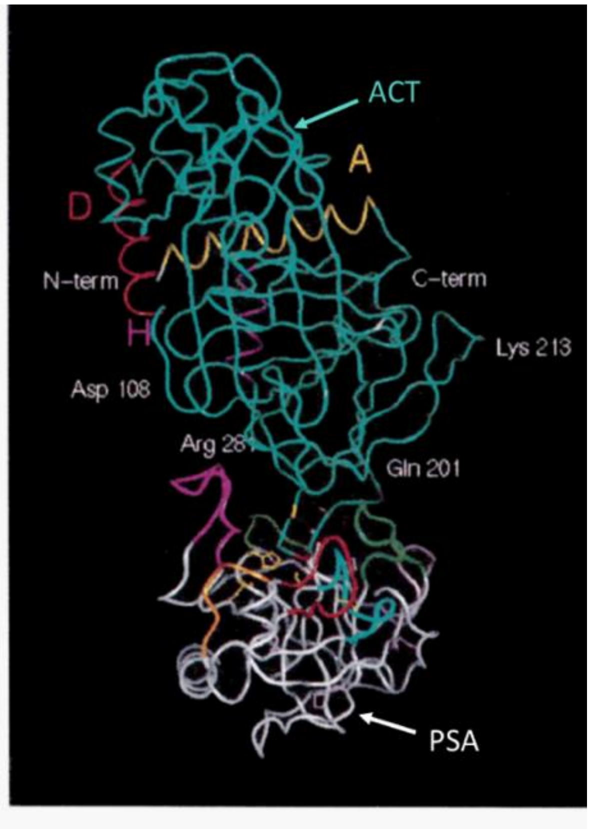

Figure S2. Prostate-specific antigen (PSA) in white complexed with alpha(1)-antichymotrypsin (ACT) in blue [15].

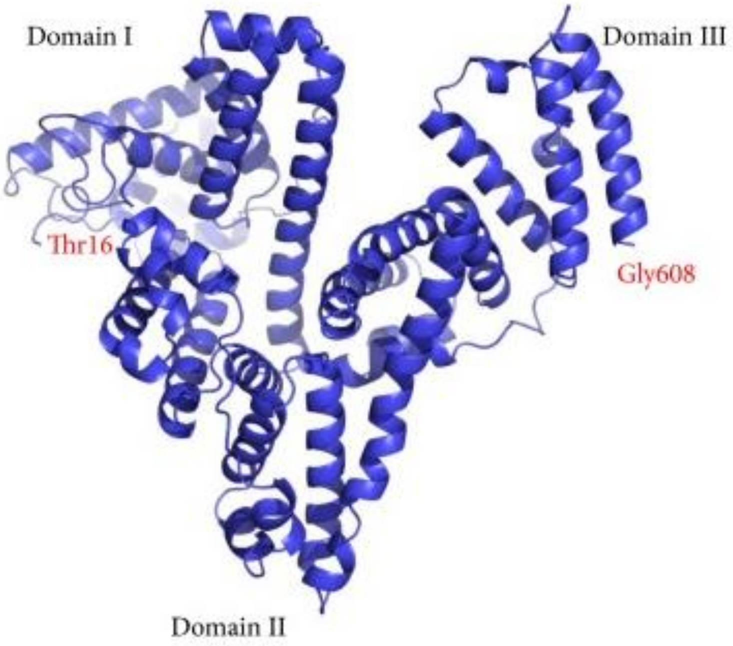

Figure S3. Alpha-fetoprotein (AFP) monomer structure model. AFP is a V-shaped protein of about 70 kDa with Stokes radius of 3.26 nm [24].

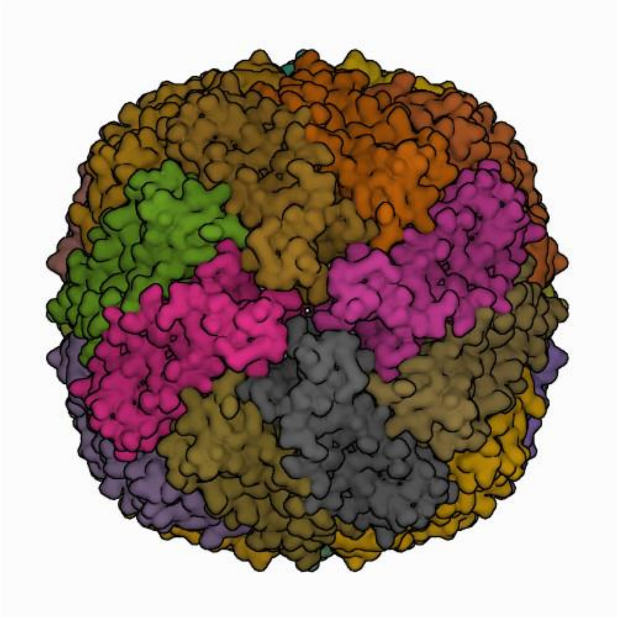

Figure S4. Cartoon representation of Ferritin, a globular heterodimeric protein of 474 kDa with hydrodynamic radius of 6 nm. PDB accession code 1FHA [25].
